# Caregivers’ motivations for using pediatric Tuina for recurrent respiratory tract infections in young children in Southern China: a qualitative study

**DOI:** 10.1186/s12906-026-05364-9

**Published:** 2026-03-31

**Authors:** Lingjia Yin, Mi Lin, Bei Chang, Darong Wu, Cecilia Stålsby Lundborg, Helle Mølsted Alvesson

**Affiliations:** 1 State Key Laboratory of Dampness Syndrome of Chinese Medicine, The Second Affiliated Hospital of Guangzhou University of Chinese Medicine, Guangzhou, China; 2 Department of Global Public Health, Karolinska Institutet, Stockholm, Sweden; 3 Center for Clinical Research, Guangdong Provincial Hospital of Chinese Medicine, Guangzhou, China; 4 The Second Clinical Medical College, Guangzhou University of Chinese Medicine, Guangzhou, China; 5 Guangdong Provincial Key Laboratory of Clinical Research on Traditional Chinese Medicine Syndrome, The Second Affiliated Hospital of Guangzhou University of Chinese Medicine, Guangzhou, China

**Keywords:** Children, Recurrent respiratory tract infections, Medical pluralism, Pediatric Tuina, Healthcare seeking patterns

## Abstract

**Background:**

Caregivers have specific concerns and preferences when seeking healthcare for their young children affected by recurrent respiratory tract infections. In China, medical pluralism is widespread. Caregivers of sick children often seek treatment from both biomedicine and traditional Chinese medicine practitioners. One traditional medicine practice is pediatric Tuina, a soft massage technique administered by trained providers, which has been used to prevent new respiratory infections. However, there is limited understanding of caregivers’ perspectives on pediatric Tuina. In this study, we aim to understand why caregivers in Southern China turn to pediatric Tuina for their children’s recurrent respiratory tract infections.

**Methods:**

A qualitative interview design was used in this study. Participants were recruited using a convenience sampling technique. Sixteen mothers from Southern China, whose children had undergone pediatric Tuina in a clinical trial for recurrent respiratory tract infections, participated in online semi-structured interviews. Informed consent was obtained by first explaining the study’s purpose to caregivers over the phone, followed by the exchange of signed printed forms via express delivery. The analysis was conducted using reflexive thematic analysis with NVivo qualitative research software. Good’s model was used as a sensitizing concept.

**Results:**

This exploratory study presents context-specific insights into caregivers’ healthcare-seeking behaviors. The overarching theme was adopting a proactive and child-centered approach in choosing the appropriate type of care for sick children, encompassing four subthemes: home-based management of early symptoms, preference for individualized care, orientation toward sustained therapeutic outcomes, and concerns regarding long-term medication exposure. The findings capture the motivations of caregivers who were predisposed to using or accepting Tuina, with limited representation of non-users.

**Conclusions:**

Caregivers choose pediatric Tuina for managing recurrent respiratory tract infections primarily due to their preference for holistic, child-centered, and less invasive care. Recognizing these caregiving values is important for informing clinician-caregiver communication and for the development of integrative approaches in pediatric healthcare.

## Introduction

 Caregivers have specific concerns and treatment preferences when seeking healthcare for their children with acute respiratory tract infections. These concerns and preferences are shaped by factors such as the child’s age, the perceived illness severity, the disruption to the family’s daily routines as well as the accessibility, quality and affordability of care [1, 2]. Preferences and practices vary by region, cultural context and over time. For instance, in rural communities in Philippines and India, caregivers prefer to try home remedies before seeking medical care, particularly when symptoms are mild [3, 4]. Nearly a decade ago, in some European countries, caregivers generally avoided self-medication, placed trust in healthcare providers and were inclined to accept antibiotic prescriptions [1, 5, 6]. Recently, in Scandinavian countries, there has been a greater acceptance of a “watchful waiting” approach, reflecting broader public health values [7]. In contrast, while caregivers in some Southern European countries have shown improved awareness of antibiotic resistance, the practice of self-prescribing antibiotics persists [8].

Caregivers may have even more concerns and different preferences when their children experience recurrent acute respiratory tract infections. Young children are vulnerable to acute respiratory tract infections, and many suffer from these infections recurrently without an organic lesion. In children under the age of 3, an average of 5 to 6 episodes per year has been reported [9, 10]. While several annual episodes of uncomplicated respiratory infections in young age are typical, particular attention should be paid to children who have unusually frequent or prolonged infections. There is currently no consensus on the definition of recurrent respiratory tract infections (RRTIs). Internationally, the most common definition of RRTIs in the literature is having an acute respiratory tract infection more than six times within one year [11, 12]. In China, the estimated annual prevalence of RRTIs among children aged 3–6 years was around 25% in both 2015 and 2019 [13, 14]. Evidence from Finland and China indicates that recurrent and prolonged respiratory tract infections in children are associated with extended symptom duration, reduced health-related quality of life in both children and caregivers, and a disproportionate burden on health care services, including increased physician visits, hospitalizations, and antibiotic use [15, 16].

Many healthcare-seeking behavior models have been used to explain care-seeking practices, such as the Health Belief Model, the Theory of Planned Behaviour and Pathway models [17]. Good’s model is one of the pathway models used to understand healthcare-seeking behavior in the context of medical pluralism [18]. In this model, there are three therapy choices: self-treatment, traditional practitioners, and biomedical practitioners. Good’s model acknowledges that people can move from one type of care to another. In this study, Good’s model was used as a conceptual framework. It provided us with insights into how caregivers, when seeking healthcare for their RRTIs-affected children, navigate between these various systems based on their cultural beliefs, personal experiences, and the available treatment options.

In China, medical pluralism is a common feature of the health care system. Biomedicine and Chinese medicine have coexisted since biomedicine was introduced to China in the middle of 19th century [19]. People are free to seek help from either biomedicine or Chinese medicine or both. In the context of medical pluralism in China, pediatric Tuina (massage) is one of the options that caregivers may choose to treat RRTIs and prevent future infections. Clinically, it has been widely used to treat common childhood conditions such as cough, diarrhea, and fever [20]. Pediatric Tuina is a manual therapy characterized by gentle pressure and swift movements on acupoints specific to children, as well as those shared with adults. It has been reported that receiving six or more sessions of pediatric Tuina within a year can serve as a protective factor for young children suffering from RRTIs [21]. Although precise data on the proportion of caregivers who have used pediatric Tuina for their children’s RRTIs is lacking, real-world outpatient consultations suggest a growing trend in its adoption.

Despite its widespread use, in-depth qualitative studies exploring caregivers’ motivations remain scarce. Gaining insight into why caregivers choose pediatric Tuina can optimize clinical treatment and health services, while also investigating the integration of Chinese medicine with biomedicine, thereby enhancing the overall effectiveness of pediatric health management. Therefore, the current study aims to examine the healthcare-seeking patterns of caregivers in Southern China to understand why they turn to pediatric Tuina for their children’s RRTIs.

## Methods

This study was designed as a qualitative interview study. We utilized semi-structured interviews and adhered to the consolidated criteria for reporting qualitative research [22]. Reflexive thematic analysis was used to analyze the data [23].

### Setting and participant selection

The participants were the caregivers of children enrolled in a clinical trial conducted in Southern China [24]. The aim of the trial was to explore the characteristics of gut microbiota in children with recurrent respiratory tract infections, before and after a month-long pediatric Tuina therapy. The trial included 20 children who met the following inclusion criteria: (i) diagnosed with RRTIs, (ii) aged between 36 and 72 months, (iii) maintained a regular diet and bowel habits, and (iv) had caregivers capable of assisting with fecal sample collection. Furthermore, none of the children had allergic conditions, like asthma or eczema, or any other illnesses that might disrupt gut microbiota, such as diarrhea. We called the caregivers to see if they would like to take part in a qualitative study. Four declined participations, mainly due to time constraints. The sixteen mothers who agreed to participate all live in Dongguan, a populous city in Guangdong province with many migrants from other provinces in China [25]. Dongguan is a relatively young city, characterized by a predominantly young workforce with generally low educational attainment [26]. All participants were mothers. Five of them were stay-at-home parents, and six resided in multigenerational households. Seven reported having a single child, while the remaining participants had two children. Among the children receiving pediatric Tuina therapy, half had no prior exposure to pediatric Tuina, whereas the other half had previously undergone the treatment.

### Data collection

The data were collected through interviews conducted to address two research questions. The findings pertaining to caregivers’ perceptions and experiences of pediatric Tuina in children with RRTIs are presented in a separate manuscript [27]. Initially, the interviews were planned to be face-to-face. However, due to Covid-19, this was not feasible, and online interviews were conducted via “Wechat” voice calls instead. All interviews were conducted one-to-one and in real time, at a time convenient for participants. Participants were encouraged to join the interview from a private setting to ensure confidentiality. In total, 16 interviews were conducted by LY between January and July, 2021. LY had been trained in qualitative research during her doctoral training and has prior experience of conducting qualitative interviews and analysis. She also has a background in Chinese medicine, specifically majoring in pediatric Tuina. She was not involved in the clinical trial in which the caregiver’s children were enrolled and did not have prior relationships with the participants. Recognizing that her professional background as a pediatric Tuina practitioner could influence the caregivers’ responses, LY introduced herself primarily as a researcher during interviews.

At the beginning of the interview, she spent some time chatting with the caregivers to establish rapport. A series of open-ended and probing questions were formulated to explore the children’s illness narratives; the caregivers’ healthcare-seeking experiences with biomedicine and Chinese medicine; their perspectives on pediatric Tuina; their encounters with pediatric Tuina; the outcomes after pediatric Tuina; and the motives behind participating in the trial. Two interviews were initially undertaken to assess the guide’s feasibility, and as only slight revisions were subsequently made for clarity, these interviews were incorporated into the analysis. The interviews, which lasted between 35 and 60 min, were recorded by a Samsung voice recorder (Huizhou Samsung Electronics Co., Ltd.). Notes were taken, both during and after the interviews, to supplement the audio recordings in analysis. The information power was deemed sufficient based on the study’s objectives, the specificity of the sample, and the quality of the dialogue [28].

### Data analysis

**Fig. 1:**
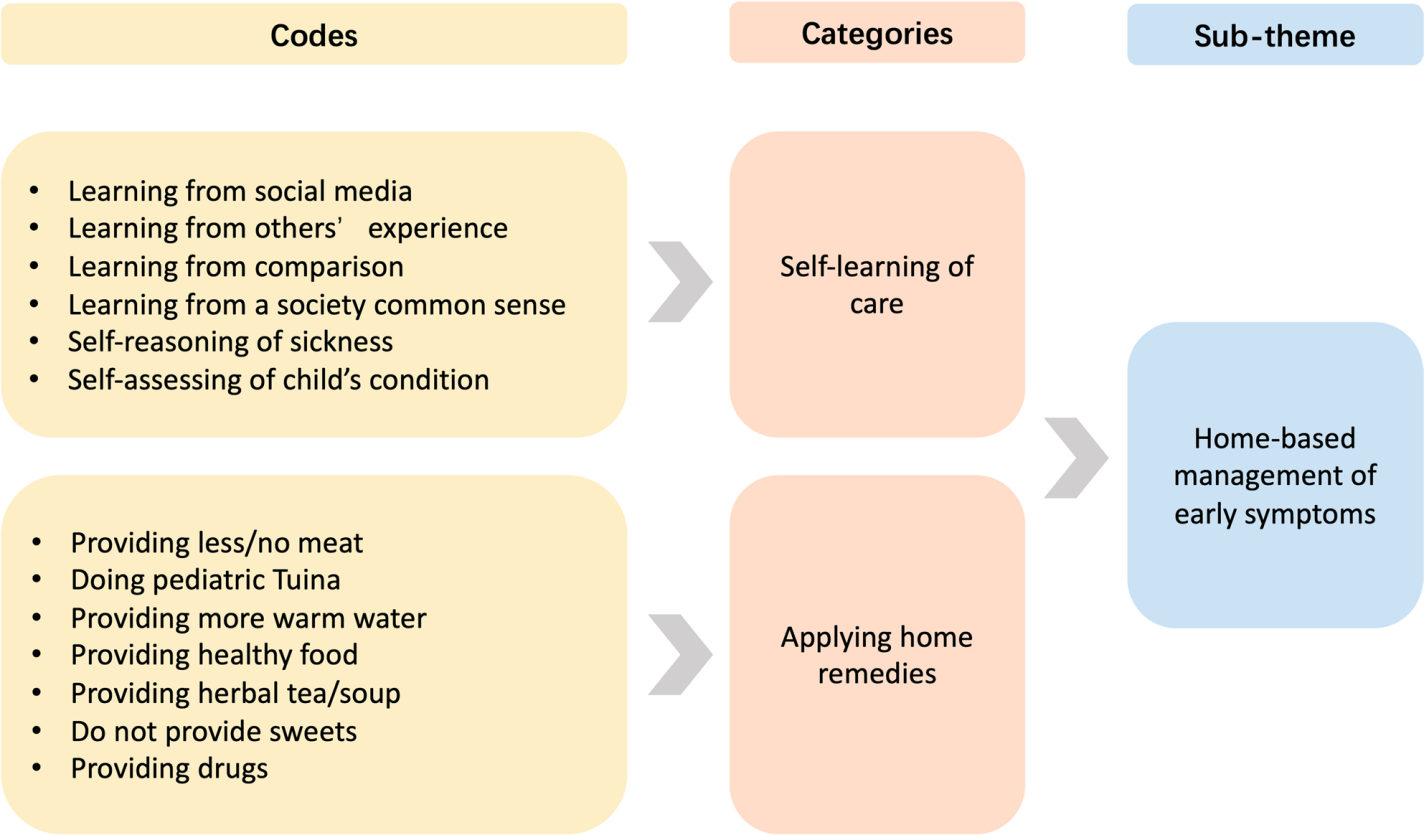
An example illustrating the transition from codes to categories and final sub-theme

This study was informed by a constructivist epistemological orientation. Meaning was understood as co-produced through participants’ narratives and the researchers’ interpretive engagement with the data. Reflexive thematic analysis was chosen to develop themes within the data [23]. The interviews were transcribed verbatim by LY and reviewed by BC. During the initial analysis period, the transcripts were read and re-read to achieve familiarity with the dataset. LY conducted line-by-line coding to remain open to all potential interpretations. Coding was conducted inductively using NVivo qualitative research software (QSR International Propriety Limited, 2019). All sixteen interviews were coded with English labels, referencing the Chinese transcripts to preserve contextual meaning [29, 30]. Following initial coding, codes were compared across interviews and iteratively clustered into broader categories based on conceptual similarity and shaped patterns of meaning. These categories represented a higher level of abstraction while still being grounded in the coded data extracts. Initial codes, categories, and candidate themes were initially discussed among LY and ML and were repeatedly reviewed against both the coded data and the full dataset. Reflexive discussions were conducted within the research team during data analysis to critically examine how the interviewer’s background might shape data interpretation. Through multiple discussions of the preliminary clusters of data, Good’s model was introduced and used as a sensitizing concept to inform the interpretation of caregivers’ healthcare-seeking behaviors [18]. Reflecting on the original aim of our study and Good’s model, we formulated the final themes into one main theme and four sub-themes. An example illustrating the transition from codes to categories and final sub-theme is provided in Fig. 1. 

### Ethical statement

This study was performed in accordance with the Declaration of Helsinki and was approved by the Ethical Committee at Guangdong Provincial Hospital of Chinese Medicine, China (YE2019-204-01) in 2019. Prior to the interview, informed consent was obtained after explaining the study’s purpose to participating mothers over the phone. Due to COVID-19 restrictions, it was difficult to meet the caregivers in person and sign the informed consent. Therefore, printed informed consent forms were sent to the participants via express delivery and returned to the researcher once they had been read and signed. Confidentiality was ensured by placing the forms in sealed envelopes, which were then handed to the courier by both the researcher and the participants. 

## Results

**Fig. 2:**
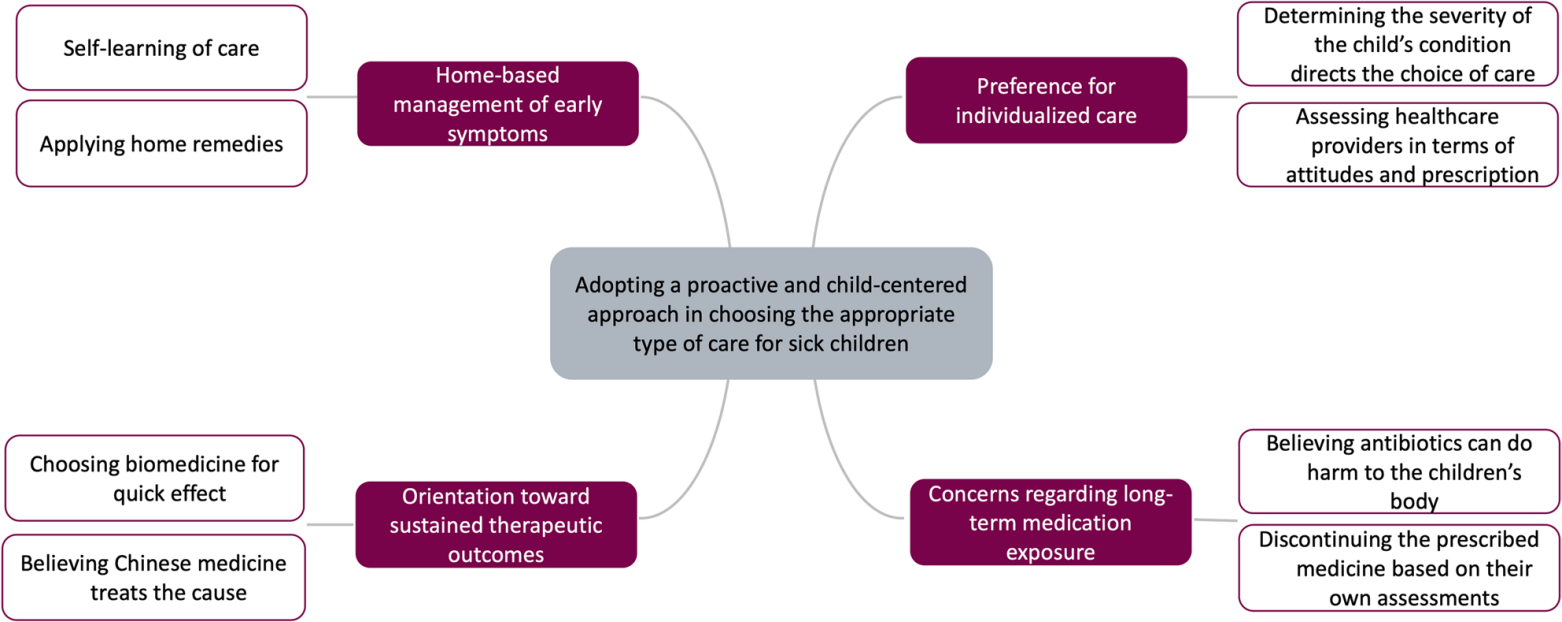
Overarching theme, sub-themes and categories

The overarching theme is “Adopting a proactive and child-centered approach in choosing the appropriate type of care for sick children” Rather than relying solely on a single form of treatment, caregivers described how they actively managed the mild symptoms at home and make thoughtful decisions based on their perceived child’s needs. Within this overarching orientation, four subthemes emerged:1. Home-based management of early symptoms, 2. Preference for individualized care, 3. Orientation toward sustained therapeutic outcomes, 4. Concerns regarding long-term medication exposure (Fig. 2).

### Subtheme 1. home-based management of early symptoms

This subtheme reflects caregivers’ tendency to initially manage their children’s mild symptoms within the home setting before seeking professional healthcare. This home-based management was manifested in two related aspects: caregivers’ self-learning of care, in which they sought information and knowledge about symptom management, and applying home remedies, where they implemented commonly used household care practices to relieve symptoms.

Self-learning of care was the main approach adopted by caregivers to manage the recurrence of acute respiratory tract infections. With the rapid development of online medical resources, the ability to learn about care practices on social media was appreciated. Mothers mentioned that they had followed WeChat public accounts related to children’s health and read their popular science articles. They extracted the knowledge that they thought would benefit their own children and applied it to the prevention and management of their children’s illness. In addition, they also talked to relatives, friends, and health care providers to obtain information about their children’s health. Some caregivers interpreted the health status of their chilren based on former illness episodes and experiences. During the interviews, mothers described their children’s recurrent illness in a way that suggested that they were not conscious of all the efforts they had made, since these were integrated in their daily lives.

*“Anyway*,* if there is anything related to children that is promoted on a public account*,* I will take a look…Sometimes she coughed severely*,* and other times the cough was not severe… Then I checked out Dr. Ding’s (pseudonym name) recommendations on the public account (a social media account)”* (ID:02, with a 4-year-old child)

Caregivers were hoping to stop the development of the sickness by applying home remedies. Most mothers reported that they would treat the child at home for mild symptoms such as sneezing, nasal obstruction and runny nose, based on what they had previously learned. The main strategy used was diet management, most commonly in the form of providing the child with a certain herbal tea or soup. Some mothers avoided protein-rich foods, fried food or sweet food during the sickness, while others offered more water to the child. In addition to diet management, mothers also commonly used pediatric Tuina and medicines that had been effective during former illness episodes.

*“Sometimes*,* he had sore throat*,* I would buy some fish mint* (*Houttuynia cordata*, *a herb native to Southeastern Asia used in Asian cuisine and medicine for its anti-viral*,* anti-inflammatory and anti-bacterial properties) and make tea for him. If it was not severe at the beginning. I would also take him to do pediatric Tuina*,* which is close by.”* (ID:03, with a 6-year-old child)

*“Initially*,* I wouldn’t take her to see a doctor. I would first make her drink more water*,* give her some vitamin C. Or sometimes*,* for the same symptoms (as the last time)*,* if the medicine prescribed by the doctor last time was effective*,* I might give her that cough medicine again. If the situation is not very serious*,* it won’t worsen further. Then I would insist on making her drink more water*,* have no meat*,* no candies nor biscuits*,* eat more fruits and applying Tuina on her back. If there is improvement with these measures*,* I would not go to the doctor.”* (ID: 09, with a 5-year-old child)

### Subtheme 2. preference for individualized care

This subtheme captures caregivers’ efforts to select care that best fits their child’s specific needs and circumstances. Caregivers described making healthcare decisions through an evaluative process that involves both assessing the child’s condition and considering whether healthcare providers offered individualized treatment approaches.

Based on their experiences of previous hospital visits, mothers developed their own health care seeking principles. Usually, they would consider the disease to be severe when the child had a cough or fever. If they considered the child to be severely sick or if they thought that the child needed nebulizer therapy, they would visit a tertiary hospital. Otherwise, they preferred to visit community hospitals, which they felt were more convenient, due to shorter distance and waiting time, and lower cost.

*“I usually go to a nearby clinic first and see how it goes. If there’s no improvement after a few days*,* I then go to a large hospital for further examination*,* including blood tests*,* to check whether it’s viral or bacterial. I still feel a bit uneasy otherwise.”* (ID: 07, with a 5-year-old child)

*“Those (community hospitals) in the village*,* are more convenient and closer. You don’t have to wait in long queues. With waiting and seeing the doctor*,* half the day is already gone. Children are already uncomfortable and going to a crowded place like a hospital feels less convenient compared to the community here.”* (ID: 06, with a 6-year-old child)

Mothers wanted health care providers to provide their children with personalized treatment. Due to the recurrence of their children’s sickness, caregivers became very experienced at critically assessing health care providers. Interestingly, when they talked about the differences between hospitals, they referred to the differences between healthcare providers’ prescription patterns. Mothers reported that since the doctors in tertiary hospitals tended to prescribe the same medicine all the time, they preferred those in the community hospitals, who did not have a fixed prescription practice. In addition, mothers described not wanting to seek help from a private clinic, as they considered the providers at most of these to be less professional for pediatric Tuina. They preferred to seek help from a qualified Chinese medicine or pediatric Tuina provider.

*“To be honest*,* I’m not a big fan of these large hospitals. Many doctors there are often busy*,* providing minimal explanations. Moreover*,* the medications they prescribe seem to follow a fixed pattern based on your symptoms. It feels like they have established a standardized template*,* and they don’t tailor their prescriptions to individual cases. It seems they apply a one-size-fits-all approach.”* (ID:05, with a 6-year-old child)

### Subtheme 3. orientation toward sustained therapeutic outcomes

This subtheme captures caregivers’ considerations about the temporal dimension of treatment outcomes when selecting healthcare for their children. Rather than focusing solely on immediate symptom relief, caregivers described weighing the perceived strengths of different medical approaches in achieving both short-term and longer-term benefits.

Caregivers believed in the existence of a cure or a “better” treatment for their sick children. Due to the recurrence of this disease, many mothers were not satisfied with the diagnosis and the treatment of each individual sickness episode. They desired a treatment that would cure the condition. Having failed to receive satisfactory health care from biomedicine alone, they were open to also testing certain traditional treatments. On the one hand, mothers chose biomedicine when they needed a quick diagnosis and quick symptom relief for a single sickness episode. On the other hand, they sought Chinese medicine to cure the cause of the recurrent illness, by adjusting the children’s constitution. Some mothers preferred to seek help from a health care practitioner who would prescribe biomedicine and patent Chinese medicine in combination or who would also administer pediatric Tuina during the early stages of an episode. However, most of the mothers were unfavourable towards herbal decoctions, which were too bitter for their children to take.

“*I am thinking*,* for recurrent respiratory infections*,* would pediatric* Tuina *help to improve the condition? It’s not necessarily about complete recovery*,* is it? Achieving complete recovery might be impossible. As long as there is improvement*,* that would be good*,* right? If it has this effect*,* then you would definitely be willing to give it a try.*” (ID:06, with a 6-year-old child)

“*After she reached the age of two*,* I began using traditional Chinese medicine. Initially*,* she was reluctant to take it*,* but later*,* she started to accept it. After a period of taking Chinese medicine*,* it seemed to have some positive effects…… When she was little (under the age of two)*,* it was tough because she couldn’t bear the bitterness of Chinese medicine.*” (ID:15, with a 4-year-old child)

### Subthemes 4. concerns regarding long-term medication exposure

This subtheme captures caregivers’ apprehension about the potential risks associated with prolonged medication use in children. These concerns were reflected both in caregivers’ beliefs about medication safety and in their medication-related decision-making.

Mothers were anxious about the long-term use of medications from both biomedicine and Chinese medicine, especially antibiotics. They formed their own views about antibiotics through self-learning from social media and their own experiences of using antibiotics on their children. When talking about antibiotics, many mothers used the Chinese word for anti-inflammatory drugs. Generally, they believed antibiotics to be harmful, because of the side effects they observed in their children, such as sweating, poor appetite, listlessness, and delayed growth. According to some, while killing the bad bacteria, antibiotics also killed the good ones, potentially leading to less appetite, lower digestion and absorption function and worse immunity. Some mothers even believed the repeated use of antibiotics to be the cause of the recurrent sickness of their children and therefore expressed a sincere desire to reduce their use. Only two mothers spontaneously mentioned concerns about antibiotic resistance. Some mothers could not give specific reasons for perceiving antibiotics to be harmful, believing this to be the case because people around them said so.

*“It’s not that we oppose taking antibiotics; when it’s necessary*,* we should definitely follow the doctor’s advice to take them. It’s just that if he frequently falls ill*,* he ends up taking antibiotics frequently*,* and it becomes a vicious cycle. Over time*,* this may lead to him remaining thin and small.”* (ID:04, with a 4-year-old child)

*“He gets sick quite frequently……That’s why we were quite interested when we saw the pediatric Tuina*,* because he has taken too many medications. His gastrointestinal system isn’t very good*,* and when he takes a lot of medicine*,* he may also vomit.”* (ID:08, 4-year-old child)

*“Antibiotics can kill bacteria*,* but they can also kill the body’s own bacteria. In other words*,* they have both benefits and drawbacks……Moreover*,* every time he has used antibiotics-like the last time he had a febrile seizure and received injections (of antibiotics) -he started to have excessive sweating*,* which lasted for quite a long time. Even now*,* he still often has episodes of profuse sweating.”* (ID:11, with a 4-year-old child)

As a result of these concerns, mothers were cautious about administering antibiotics to their children, even when they were prescribed by health care practitioners. Sometimes mothers would even take the decision to stop the treatment. This was also the case for Chinese medicine.

*“Later*,* I went to see a traditional Chinese medicine practitioner. After receiving the prescribed herbal medicine*,* I researched each herbal name one by one. I felt like it didn’t quite suit her. But I still gave her the first dose*,* and after taking it*,* I didn’t see any improvement. The doctor prescribed three doses in total*,* but I didn’t give her the last two.”* (ID:01, 3-year-old child)

## Discussion

Understanding the reasons caregivers utilize pediatric Tuina for children with recurrent respiratory tract infections is essential for improving communication between health care practitioners and caregivers, customizing treatments, honoring cultural beliefs, and advancing research in pediatric care.

In the context of medical pluralism, Good’s model [18] offered a framework for understanding caregivers’ healthcare seeking behaviors. In the model it is emphasized that people rarely follow a strictly linear path; instead, they often shift between different care systems depending on how they perceive the illness and the type of care they consider most appropriate. It also underscores the influence of “significant others” - the relatives and friends who play a substantial role in shaping individual decision-making. In this study, the patterns of seeking children’s health information from friends and relatives within the first sub-theme, “Home-based management of early symptoms”, as well as the patterns of choosing biomedicine for rapid symptom relief and seeking Chinese medicine to address the root cause within the third theme, “Orientation toward sustained therapeutic outcomes”, confirmed the central tenets of Good’s model. However, the model alone is insufficient for fully capturing caregivers’ practices. Through this study, we identified additional pathways that shape healthcare choices before therapy selection, including self-learning of care through social media, preferences for individualized healthcare and concerns about long-term medication use.

The recurrence of acute respiratory tract infections, combined with the desire to be good parents, motivated caregivers to manage their child’s illness proactively. They hoped to stop the illness at an early stage and feel more in control to avoid additional hospital visits. Caregivers preferred self-learning about care options during the early stages of illness. This is in line with findings from many contexts, particularly when symptoms are mild [3, 4]. During the home care of mild symptoms, the caregivers came to learn and apply pediatric Tuina. It has been found in previous studies that caregivers can administer pediatric Tuina at home [31, 32].

Online health information emerged as the primary resource for caregivers to glean knowledge about how to handle the recurrent episodes at home during the early stage of illness. In a systematic review, the prevalence of parental online health information seeking was reported to be 52%-90% [33]. In some high-income countries, caregivers have been seen to approach online health information cautiously, with a study in Switzerland revealing that 91% of parents harbored skepticism about its accuracy, and 67% sought guidance from pediatricians [34]. They viewed online information not as a substitute for healthcare services but as a supplementary tool [35, 36]. Conversely, in Australia, only half of caregivers took steps to verify the reliability of online publishers before accepting information [37]. To date, no studies have explored how Chinese caregivers utilize online health information for their sick children. The extent of their caution in approaching this information also remains unclear and should be further studied.

Caregivers’ inclination to seek other health care due to doctors’ rigid prescription practices highlights their preference for personalized healthcare. Previous studies have shown that patients and caregivers appraise healthcare not solely by clinical outcomes, but also by the extent to which it feels individualized, attentive, and congruent with their expectations and values [38]. This holistic evaluation drives satisfaction and adherence [39]. From a practitioner’s viewpoint, adhering to fixed prescriptions aligns with guidelines. However, in this study, caregivers perceived this as a lack of quality care. Within pluralistic healthcare systems, Chinese medicine is frequently perceived as offering a more holistic and individualized approach that differs through syndrome differentiation. This is a feature that resonates strongly with caregivers’ expectations of tailored care [40]. While this individualized logic poses challenges for standardization and strict guideline adherence [41, 42], such concerns were largely irrelevant to caregivers, who prioritized the experiential sense of being treated as unique cases. Consistent with findings from a systematic review about the views on Chinese medicine [43], caregivers in this study believed that Chinese medicine is beneficial for chronic disease, and for the symptoms that they perceived as insufficiently managed by biomedicine. This turn toward Chinese medicine does not signal a rejection of biomedicine, but rather a strategic health-seeking response aimed at securing more lasting relief and a form of care that resonates with caregivers’ understanding of their children’s needs.

The recurrence of the sickness significantly influenced mothers’ attitudes towards prescribed treatment, particularly antibiotics. The anxiety of using medication largely motivated the caregivers to seek help from non-pharmaceutical therapies. It is crucial to acknowledge that children who suffer from RRTIs use antibiotics more often than children who do not [15]. Their caregivers have therefore had more opportunities to observe their children’s reactions to these medications. Consequently, they are more likely to notice any side effects. In this study, some mothers even expressed the view that taking antibiotics too often leads to recurrence of sickness. Studies in China have indicated that children who receive antibiotics more than three times in a year face a higher risk of RRTIs [44, 45]. A study in Southern China showed that controlling antibiotic use significantly reduced infections rates in children under three years old [46]. Therefore, it is understandable that caregivers may feel anxious about the long-term use of antibiotics and healthcare practitioners could address these concerns with caregivers before prescribing these medications.

### Clinical implications and recommendations

These findings have several implications for clinical practice. First, healthcare professionals should recognize caregivers as active decision-makers who prioritize both immediate symptom relief and long-term well-being for their children. Acknowledging caregivers’ concerns about medication burden and treatment safety may help build trust and improve communication. In clinical practice, healthcare providers could enhance communication by actively inviting caregivers to express their worries, offering information about treatment benefits and potential risks. Second, clinicians could benefit from proactively discussing integrative care options, including pediatric Tuina, when appropriate, rather than framing such practices as alternatives outside the formal healthcare system. Clear guidance on indications, limitations, and safe integration with biomedical treatments may support more coordinated care and reduce caregivers’ uncertainty. In practice, integrating pediatric Tuina into standard care pathways could involve incorporating evidence-based recommendations into clinical guidelines and promoting interdisciplinary communication within healthcare teams. Third, clinicians should recognize that caregivers often consult online information before seeking care and may arrive with pre-formed expectations. Encouraging open discussion about online information use and guiding caregivers toward reliable sources may help reduce misinformation and support safer home management. Developing accessible, evidence-based online resources tailored to caregivers could further align clinical guidance with caregivers’ proactive care-seeking behaviors. Finally, adopting child-centered and family-centered communication strategies, such as explicitly acknowledging individual child characteristics and clearly communicating how guideline-based treatments can be adapted to the child’s specific context, may help clinicians better understand caregivers’ concerns and priorities. Such mutual understanding can support more coordinated care and may ultimately improve adherence and satisfaction.

### Reflexive considerations and limitations

There were several reflexive considerations and limitations in this study. Firstly, caregivers were recruited exclusively from a clinical trial on pediatric Tuina, which may have introduced a degree of selection bias, as caregivers who agreed to participate were likely to have prior exposure to Tuina and generally a positive attitude toward this therapy. Consequently, the motivations explored in this study primarily reflect the perspectives of caregivers who were already inclined to use or accept Tuina, rather than those of non-users or sceptical caregivers. Second, the study was conducted in a single geographical area (Dongguan), which may limit the transferability of the findings to other regions where caregivers have higher education level or different cultural practices. Third, all interviews were conducted via voice-only online calls, which may have constrained rapport-building and limited access to non-verbal cues that can enrich the qualitative data. Participants’ responses may also have been influenced by the home-based interview setting, including the presence of family members or interruptions, which could affect the depth or openness of discussion. These contextual influences were considered during analysis through reflexive discussions within the research team. Another limitation is the absence of fathers’ perspectives. While mothers typically spend more time with their children and offer detailed insights, excluding fathers’ experiences may overlook valuable perspectives. Future research could extend this work by including caregivers with no prior experience of Tuina, particularly fathers and other family caregivers, and by recruiting participants from more diverse geographical and sociocultural settings to capture a broader range of healthcare-seeking motivations.

## Conclusions

This study highlights caregivers’ perspectives on managing RRTIs in young children, with particular focus on pediatric Tuina as an integrative care option. Key findings indicate that caregivers’ decisions are shaped by both perceived treatment effectiveness and the desire for child-centered care, and that they are concerned about the potential harms of long-term medication use. Clinically, acknowledging these concerns, proactively discussing integrative care options, and providing clear guidance on safe integration with biomedical treatments can enhance trust, improve communication, and support shared decision-making. Future research should expand the scope by including fathers, caregivers who do not use Tuina, and multicenter designs to validate findings and inform evidence-based integration of pediatric Tuina into standard care pathways.

## Data Availability

The anonymized transcripts may be obtained from the first author (LY) if a reasonable request is made.

## Abbreviations

RRTIs: Recurrent respiratory tract infections
HRQOL: Health related quality of life
